# Absence of Cyanotoxins in Llayta, Edible Nostocaceae Colonies from the Andes Highlands

**DOI:** 10.3390/toxins12060382

**Published:** 2020-06-09

**Authors:** Alexandra Galetović, Joana Azevedo, Raquel Castelo-Branco, Flavio Oliveira, Benito Gómez-Silva, Vitor Vasconcelos

**Affiliations:** 1 Laboratorio de Bioquímica, Departamento Biomédico, Facultad Ciencias de la Salud, and Centre for Biotechnology and Bioengineering, CeBiB, Universidad de Antofagasta, Antofagasta 1270300, Chile; alexandra.galetovic@uantof.cl (A.G.); benito.gomez@uantof.cl (B.G.-S.); 2 Centro Interdisciplinar de Investigação Marinha e Ambiental- CIIMAR, 4450-208 Matosinhos, Portugal; joana.azevedo@ciimar.up.pt (J.A.); raquel.castelobranco.12@gmail.com (R.C.-B.); oliveira_flavio@outlook.pt (F.O.); 3 Faculdade de Ciências da Universidade do Porto, 4169-007 Porto, Portugal

**Keywords:** cyanobacteria, cyanotoxins, Llayta, microcystin, *Nostoc*

## Abstract

Edible Llayta are cyanobacterial colonies consumed in the Andes highlands. Llayta and four isolated cyanobacteria strains were tested for cyanotoxins (microcystin, nodularin, cylindrospermopsin, saxitoxin and β-N-methylamino-L-alanine—BMAA) using molecular and chemical methods. All isolates were free of target genes involved in toxin biosynthesis. Only DNA from Llayta amplified the *mcy*E gene. Presence of microcystin-LR and BMAA in Llayta extracts was discarded by LC/MS analyses. The analysed Llayta colonies have an incomplete microcystin biosynthetic pathway and are a safe food ingredient.

## 1. Introduction

Members of the cyanobacteria genera *Microcystis, Anabaena, Oscillatoria, Planktothrix* and *Nostoc* are able to synthesize cyanotoxic secondary metabolites such as microcystin, nodularin, cylindrospermopsin, saxitoxin or β-N-methylamino-L-alanine (BMAA), a non-proteinaceous amino acid form [1,2,3,4,5]. Thus, any food or ingredient originating from cyanobacterial biomass and destined for human consumption must be seriously scanned for the presence of these toxins [6]. This is particularly important in the case of Llayta. Llayta is a foodstuff consumed by rural Andean communities since pre-Columbian times [7,8]. The vernacular name Llayta identifies the dry biomass of macro colonies of a filamentous cyanobacterium that grows at Andean wetlands over 3000 m of altitude. The cyanobacterium has been isolated from Llayta colonies (denominated *Nostoc* sp. strain LLA-15) and its taxonomy and biochemical composition has been previously reported [9,10]. After harvesting and sun drying, Llayta can be purchased today at food markets in Tacna (southern Peru) and Arica and Iquique (northern Chile) and used for the preparation of local dishes [11]. Studies on the biochemical composition of Llayta showed that 60% of total amino acids were essential amino acids for humans and 32% of total fatty acids were polyunsaturated fatty acids [10]. To date, no data on the potential for toxin producing in Llayta have been reported, although there are no epidemiological reports that show that consumption of Llayta may cause human illnesses. Based on this body of evidence, we hypothesize that Llayta available at southern Peruvian and northern Chilean food markets for human consumption is a biomass free of cyanotoxins. To support this hypothesis, we provide genetic and analytic evidence.

## 2. Results and Discussion

**Figure 1:**
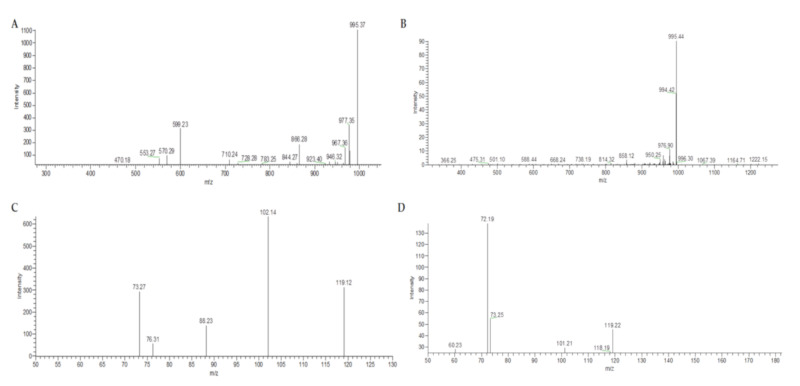
Mass spectra for standards MC-LR and BMAA and Llayta extracts. (**A**): Microcystin LR standard (SIGMA). (**B**): Llayta extract in 50% methanol. (**C**): BMAA standard (SIGMA). (**D**): Llayta hydrolyzate in 50% methanol.

The target genes *mcyA*, *cyrJ*, *anaC*, *sxtA* and *sxtG* were not amplified in any sample. The *mcy*A gene can be used in the early warning of cyanotoxins with good certainty, but according to Jasser et al. (2017) [12], they preferentially amplify *Microcystis* and *Planktothrix* genera. On the other hand, the *mcy*E gene was only amplified from Llayta DNA. Since *mcy*E gene primer sequences are found in several microcystin producer genera, the detection of this gene in opposition to the absence of *mcy*A gene is reasonable. However, the gene *mcyE* was amplified from natural Llayta DNA only. Interestingly, DNA from strain LLA-15 did not amplify the *mcyE* gene, even though LLA-15 was isolated from the dry biomass of Llayta. The *mcyE* amplicon from Llayta DNA was recovered and sequenced, and the BLASTn at the NCBI data bank showed 96% identity with gen *mcyE* from *Nostoc* sp. 152, a known microcystin-producing strain [13]. Then, the amplified *mcy*E gene could belong to other microcystin-producing cyanobacteria present in Llayta colonies; in fact, the metagenomics of the closely associated microbiome of edible Llayta colonies show an abundance of bacteria from the phylum Proteobacteria and several cyanobacterial phyla dominated by *Nostoc* (38%), *Anabaena* (25%) and *Nodularia* (20%), with minor contributions of *Microcystis* and *Cylindrospermopsis* (unpublished data). In addition, it is important to highlight that molecular screening for cyanotoxins only gives us preliminary data for potential production, being crucial to confirm the data by analytical methodologies. For that reason, after molecular screening, the MC-LR evaluation by HPLC-PDA was performed in Llayta biomass extracts. MC-LR standard injection showed a peak at 8.6 min with an absorption maximum at 238 nm. The Llayta HPLC chromatogram showed a weak signal at 8.79 min with absorption maxima at 238 nm and 390 nm. Then, it was decided to conduct a MS for this weak signal, since this might be evidence for the presence of microcystin in the Llayta extract. As expected, the standard MC-LR microcystin LR showed an RT of 7.21 min and mass fragments of 995.37-977.36-866-599 m/z (Figure 1A). The Llayta sample in LC-MS did not show a signal at RT 7.21 min. The LC signal region with RT 6–7 min was analysed by MS, and did not show the fragment pattern expected for microcystin LR. Together, the evidence supports the notion that Llayta biomass does not contain a microcystin LR-like toxin (Figure 1B). Interestingly, the Llayta LC chromatogram showed a strong signal at approximately 14 min, which becomes a subject for future work, in order to discover which molecules are there.

Dominant microcystins produced by *Nostoc* strains are DMadda or ADMadda variants, like the toxic [ADMAdda^5^] MC-LR (m/z 1009), the [DMAdda^5^] MC-LR (m/z 981) and the [D-Asp^3^, ADMAdda^5^] MC-LR (m/z 1023) [14]. The Llayta spectra (Supplementary Materials: Figures S1–S6) obtained did not contain any of the fragments reported for this MC-LR variants or for DMadda or ADMadda moieties due to the absence of the diagnostic ions at m/z 553 [Mdha-Ala-Leu-MeAspArg + H^+^] and m/z 627 [Arg-ADMAdda-Glu + H^+^]. DMAdda (m/z 121.06) and ADMAdda (m/z 163.08) specific fragment ions were also absent. The absence of all those reported [15,16] characteristic fragments confirms the safety of Llayta as a food ingredient with respect to cyanobacteria toxins.

MC-LR biosynthesis is dependent on the expression of a gene cluster that includes genes *mcy*A and *mcy*E and cyanobacteria are not able to produce the toxin if one or more of these genes are lost during evolutionary processes, as has been reported for *Microcystis aeruginosa*, *Microcystis viridis*, *Microcystis wesenbergii* and *Microcystis ichtyobable* [17,18,19,20]. A similar explanation can be inferred from our results, in which Llayta DNA amplified the gene *mcy*E but not the gene *mcy*A, and MC-LR was not present in Llayta extracts.

The LC chromatogram and MS spectrum for standard BMAA (Figure 1C) showed that BMAA migrated with an approximate RT of 5 min and yielded fragments of 119-102-88-76-73 m/z, in agreement with the literature [21,22,23]. In contrast, the Llayta hydrolysate showed a weak signal with an RT 5.5 min (Figure 1D), and its MS spectrum showed a different fragment pattern to that of standard BMAA.

As has been previously documented, cyanobacteria food supplements may be contaminated with cyanotoxins, either microcystins [24] or anatoxin-a [25], and the monitoring of these toxins is fundamental to prevent human intoxications. In our work, extracts of Llayta were analysed and did not show the presence of cyanotoxins. However, these results need further work to confirm the presence or absence of cyanotoxins in cyanobacterial samples used for human consumption, from different geographical regions in Peru and northern Chile, as well the seasonal effect on cyanotoxin production.

## 3. Conclusions

The results support the hypothesis that the Llayta biomass analysed does not contain MC-LR or BMAA toxins, and it can be considered a safe ingredient for human consumption.

## 4. Materials and Methods

To evaluate whether Llayta is a safe ingredient for human consumption, we evaluated the presence of target cyanotoxin genes in the genomes of Llayta (dry biomass) and four strains of cyanobacteria (LLC-10, LLA-15, CAQ-15, LCHI-10) isolated from various water bodies in northern Chile. Additionally, the presence of microcystin-LR (MC-LR) and BMAA was evaluated by HPLC-MS in the dried biomass of Llayta. Genomic DNA was extracted and purified from all isolates and Llayta (dry biomass) using the PureLink Genomic DNA Mini Kit (Invitrogen, USA), according to the manufacturer’s instructions. Table 1 shows the target genes (*mcy*A, *mcy*E*, cyr*J, *ana*C*, sxt*A and *sxt*G) and the primers used for PCR. The cyanobacterial strains *Aphanizomenon gracile* LMECYA-040, *Anabaena* sp. LEGE X-002, *Cylindrospermopsis raciborskii* LEGE 97047 and *Microcystis aeruginosa* LEGE 91339 were used as positive control for saxitoxin, anatoxin, cylindrospermopsin and microcystin, respectively, and obtained from Blue Biotechnology and Ecotoxicology Culture Collection (LEGE Culture Collection) of CIIMAR/University of Porto.

After molecular screening of the samples, the dry Llayta biomass was also examined by liquid chromatography coupled with mass spectrometry (LC-MS) in order to confirm the presence of MC-LR and BMAA. One gram of Llayta (dry biomass) was macerated with 8 mL of methanol 75% in liquid nitrogen. After two hours, 12 mL MeOH 75% was added and the methanolic suspension (20 mL) was sonicated (5 times, 60 s, 60 Hz) on ice to extract the intracellular toxins. After centrifugation (4000× *g* for 2 min, at 4 °C), the supernatant was recovered and concentrated in a rotary evaporator. This residue was suspended in 20 mL MeOH 75%, sonicated, centrifuged and evaporated as before. The final residue was dissolved in 50% (v/v) methanol and analysed by LC-MS to detect microcystin. To detect the presence of BMAA by LC-ESI-MS/MS, the resulting pellet from the maceration of Llayta was subjected to acid hydrolysis dissolving it in 1 mL 6M HCL at 110 °C for 24 h [21].

The LC-MS system used to identify and quantify MC-LR in Llayta was a Liquid Phase Chromatograph Finnigan Surveyor (Thermo Scientific, San Jose, CA, USA), coupled with a spectrometry detector (MS Mass LCQ FleetTM ion trap), with electrospray interface (ESI), including a Surveyor LC pump, a Surveyor auto sampler and a Surveyor photoelectric diode array detector (PDA). The program used for data acquisition and processing was XcaliburTM version 2 (Thermo Scientific, San Jose, CA, USA). The mass spectrometer was operated in full scan mode. The capillary voltage and tube lens were maintained at 22 and 120 kV, respectively; the spray voltage was 4.5 kV. Nitrogen was used as a sheath and auxiliary gas. The sheath gas flow rate was set at 80 (arbitrary units) and the auxiliary gas at 10. The capillary temperature was held at 350 °C. Helium was used as a collision gas in the ion trap at a pressure of 3 bar. Separation was achieved on C18 Hypersil Gold column (100 × 4.6 mm I.D., 5 μm, Thermo Scientific, Waltham, MA, USA) kept at 25 °C, with a flow rate of 0.7 mL/min. The injected volume was 10 μL in loop partial mode. Samples were injected in positive polarity mode, in Full scan (270–2000 m/z). The standards and samples were injected in duplicate and a blank and two standards of different concentration were introduced at each set of 6 samples. The standard solution of MC-LR was purchased from DHI LAB Products (Hørsholm, Denmark, Batch nº MCLR-110), with a concentration of 11.026 μg/mL. The system was calibrated using seven dilutions of the standard solution of MC-LR (between 8.5 and 180 μg/L) diluted in 50% acetonitrile (ACN). A gradient elution was used with mobile phase A (ACN) and B (water), both acidified with 0.1% formic acid (55% A and 45% B at 0 min, 90% A and 10% B at 12 min, 100% A at 12.5 min, 100% A at 15 min, 45% A and 55% B at 15.01 and 25 min). Under these conditions the MC-LR retention time (RT) was 7.21 min and the LOD and LOQ were 5.7 μg/L and 8.5 μg/L, respectively. Samples were analysed using the mass-to-charge ratio (m/z) transition of 995 > 599, at 35 eV collision energy. The MC-LR transition was monitored for 1 microscan time. The precursor ion (m/z 995) and MC-LR reference fragment ions with m/z values of 375, 553, 599, 866 and 977 were monitored in the MS/MS mode, in order to validate the presence of the toxin. To assess the presence/absence of other microcystin variants, precursor ions of [DMAdda^5^] MC-LR (m/z 981), [ADMAdda^5^] MC-LR (m/z 1009) and [D-Asp^3^, ADMAdda^5^] MC-LR (m/z 1023) were searched in the MS/MS obtained spectra. Diagnostic ions at m/z 553 [Mdha-Ala-Leu-MeAspArg + H^+^] and m/z 627 [Arg-ADMAdda-Glu + H^+^] were also scanned, as well as DMAdda (m/z 121.06) and ADMAdda (m/z 163.08) specific fragment ions. The qualitative analysis of BMAA in Llayta hydrolysates was done by LC-ESI-MS/MS as described above with the following modifications: the capillary voltage and tube lens were maintained at 33 and 115kV/a, respectively; the spray voltage was 5 kV; nitrogen was used as a sheath and auxiliary gas at a flow rate of 60 (arbitrary units) and the auxiliary gas at 20; the column used was a HILIC (100 × 4.6 mm I.D., 2.6 μm, Phenomenex, USA) kept at 40 °C, with a flow rate of 0.5 mL/min; samples were injected in positive polarity mode, in full scan (50–500 m/z); the standard solution was a mixture of BMAA and L-2, 4-Diaminobutyric acid dihydrochloride (DAB) from Fluca (Batch nº 0001418988), with a concentration of 0.145 μg/g; a gradient elution was used with mobile phases A (MeOH) and B (water), both acidified with 0.1% formic acid (90% A until 10 min, 60% A until 20 min, 50% A until 26 min), returning the start conditions and equilibrating 10 min and finally, samples were analysed using the mass-to-charge ratio (m/z) of 119 at 20 eV collision energy.

## Figures and Tables

**Table 1:** Primers used to show the presence/absence in Llayta samples of target genes required for toxin biosynthesis.

Target Gene	Primer Pair	Target Group Producers	Size (bps)	References
*mcy*A	*mcy*A-Cd1F/*mcy*A-Cd1R	Microcystin	297	[26]
*mcy*E	HEPF/HEPR	Microcystin and nodularin	472	[27]
*cyr*J	cynsulF; cylnamR	Cylindrospermopsin	586	[28]
*ana*C	*ana*C-genF/*ana*C-genR	Anatoxin	366	[28]
*sxt*A	*sxt*AF/*sxt*AR	Saxitoxin	683	[29]
*sxt*G	*sxt*GF/*sxt*GR	Saxitoxin	893	[29]

## Supplementary Materials

The following are available online at https://www.mdpi.com/2072-6651/12/6/382/s1, Figure S1. Extracted Ion Chromatogram and spectrum of Blank solution (methanol 50% + 0.1% Formic acid), Figure S2. Extracted Ion Chromatogram and Full MS^2^ spectrum of MC-LR Standard (20 ppb) at 3.01 min, Figure S3. Extracted Ion Chromatogram and Full MS^2^ spectra of Llayta extract at *m*/*z* 995.50, Figure S4. Extracted Ion Chromatogram and Full MS^2^ spectra of Llayta extract at *m*/*z* 981, Figure S5. Extracted Ion Chromatogram and Full MS^2^ spectra of Llayta extract at *m*/*z* 1009, Figure S6. Extracted Ion Chromatogram and MS^2^ spectra of Llayta extract at *m*/*z* 1023, Figure S7. Extracted Ion Chromatogram and MS^2^ spectrum of Llayta extract at *m*/*z* 121.06, Figure S8. Extracted Ion Chromatogram and MS^2^ spectrum of Llayta extract at *m*/*z* 163.07.
