# Supplementary material for: Absence of Cyanotoxins in Llayta, Edible Nostocaceae Colonies from the Andes Highlands
Source: Toxins (Basel). 2020 Jun 9;12(6):382. doi: 10.3390/toxins12060382 (PMC7354591; doi:10.3390/toxins12060382)
Supplement: Supplementary file 1 [file toxins-12-00382-s001.pdf]

Supplementary Materials: Absence of Cyanotoxins in Llayta, Edible Nostocaceae Colonies from the Andes Highlands

Alexandra Galetović, Joana Azevedo, Raquel Castelo-Branco, Flavio Oliveira, Benito Gómez-Silva and Vitor Vasconcelos

To assess the presence/absence of other microcystin variants, precursor ions of [DMAdda^5] MC-LR (m/z 981), [ADMAdda^5] MC-LR (m/z 1009) and [D-Asp^3, ADMAdda^5] MC-LR (m/z 1023) were searched in the MS/MS obtained spectra. Diagnostic ions at m/z 553 [Mdha-Ala-Leu-MeAspArg + H$^+$] and m/z 627 [Arg-ADMAdda-Glu + H$^+$] were also scanned, as well as, DMAdda (m/z 121.06) and ADMAdda (m/z 163.08) specific fragment ions.

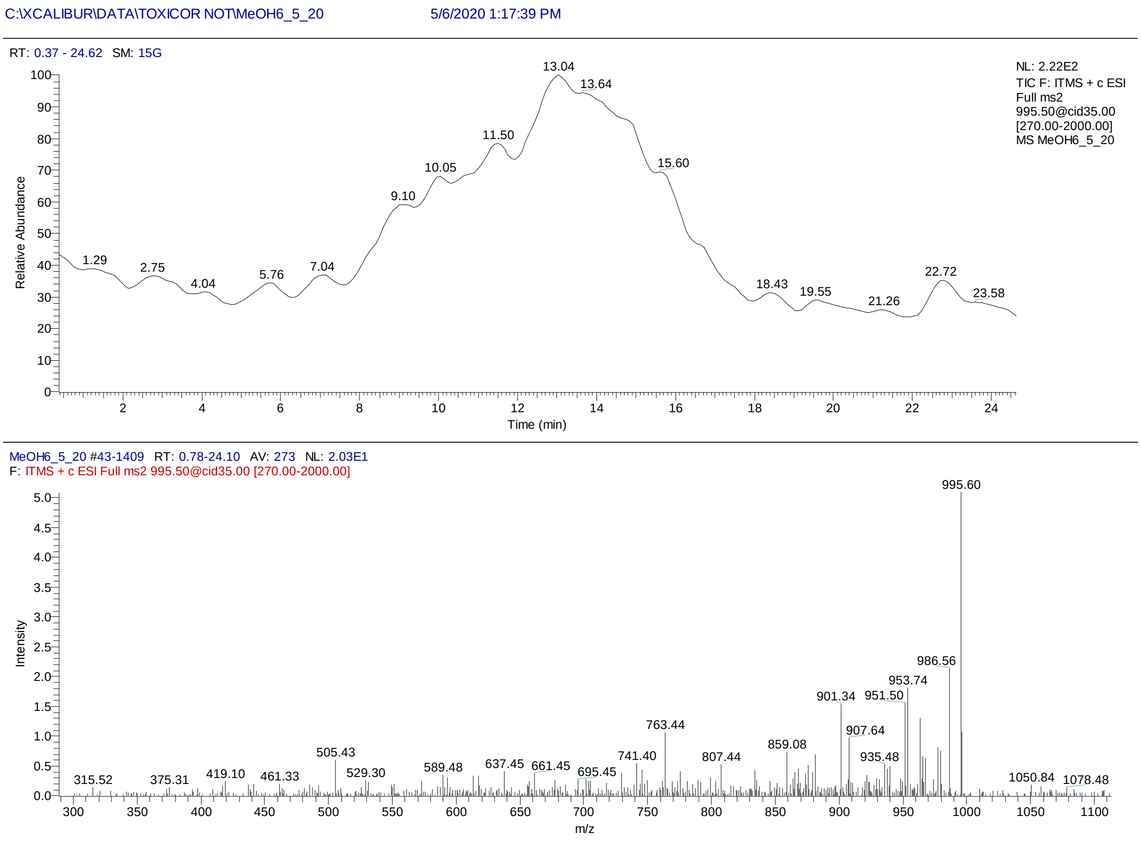

Figure S1. Extracted Ion Cromatrogram and spectrum of Blank solution (methanol 50% + 0.1% Formic acid).

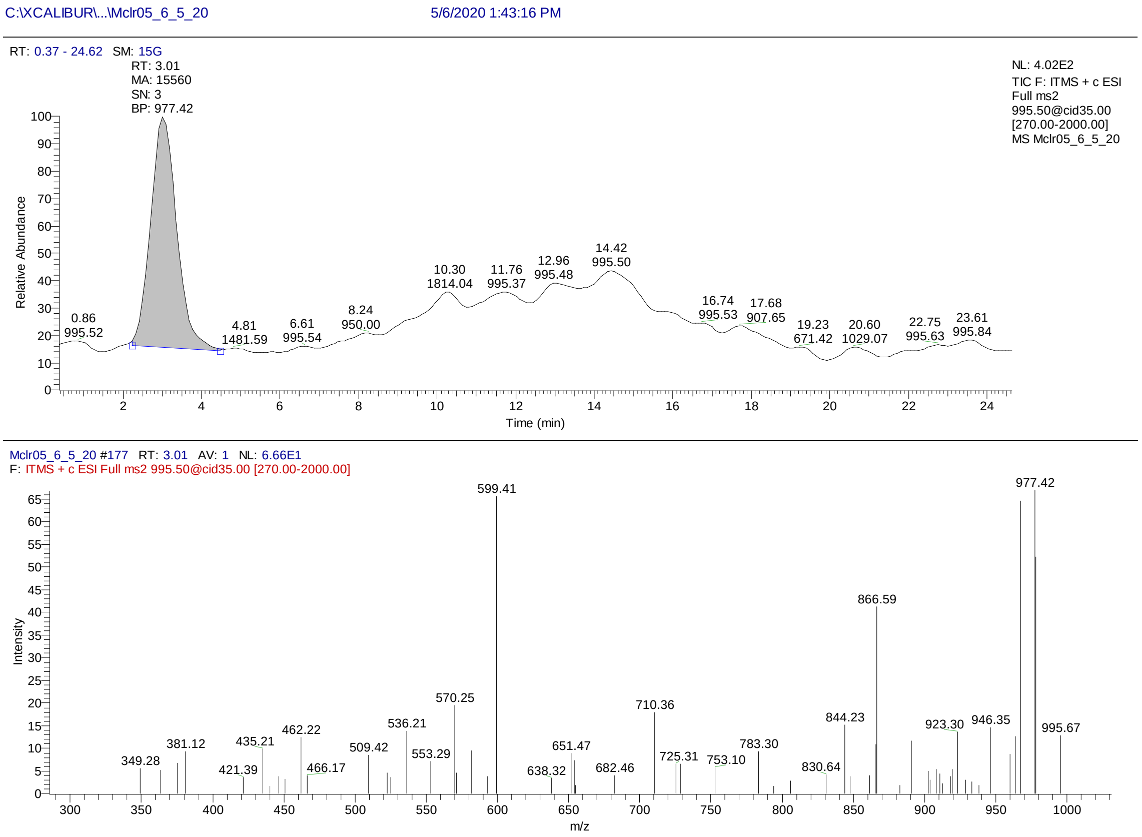

Figure S2. Extracted Ion Cromatrogram and Full MS² spectrum of MC-LR Standard (20 ppb) at 3.01 min.

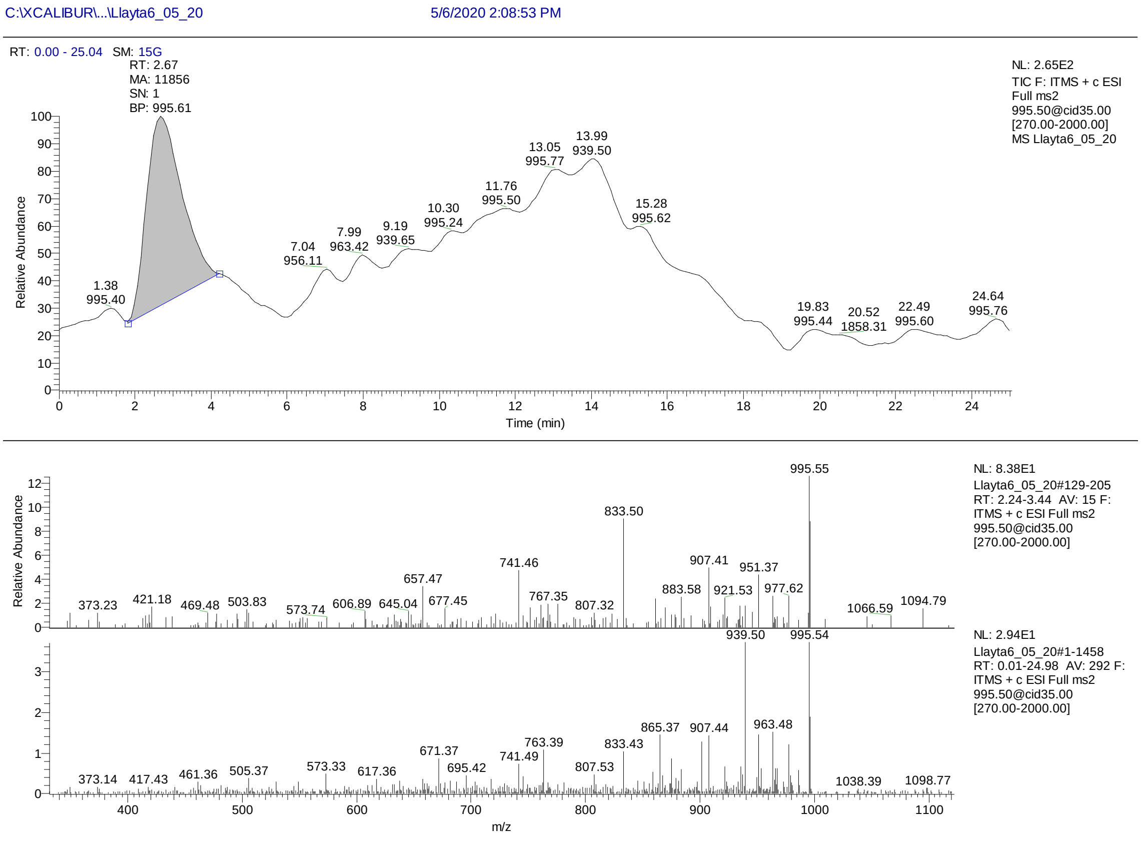

Figure S3. Extracted Ion Cromatrogram and Full MS2 spectra of Llayta extract at m/z 995.50.

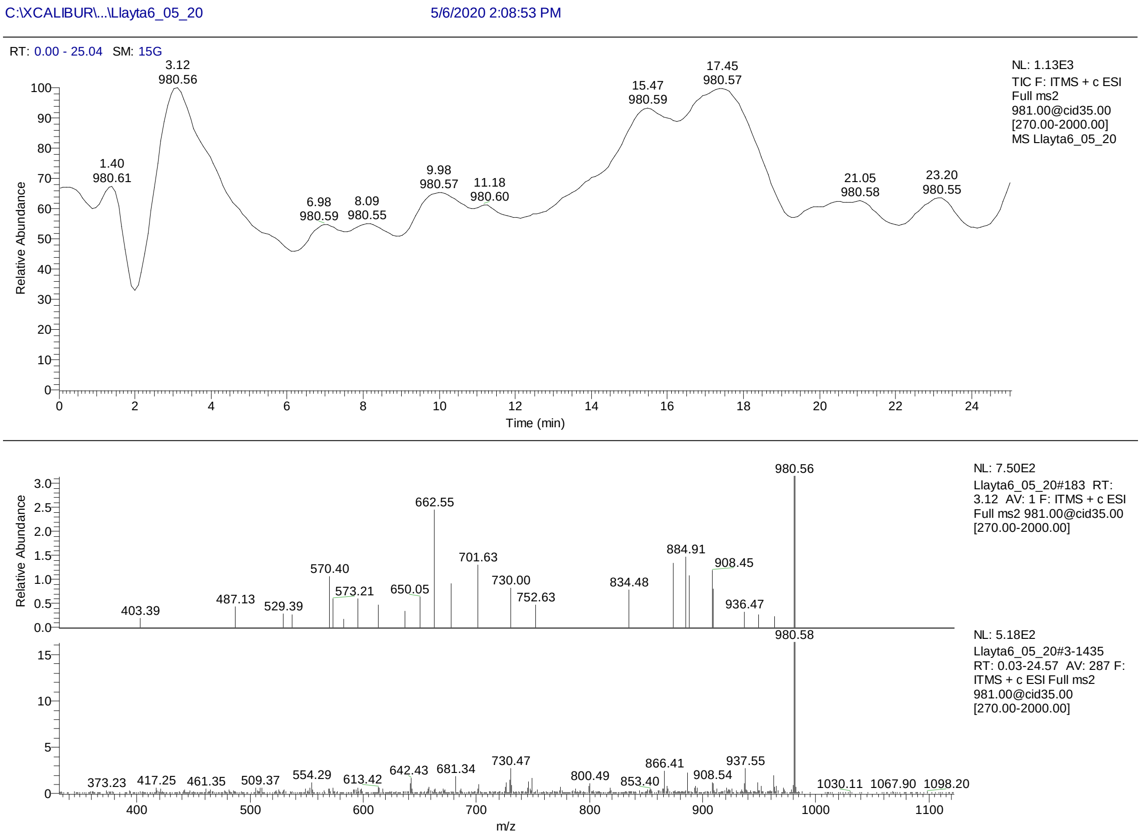

Figure S4. Extracted Ion Chromatogram and Full MS^2 spectra of Llayta extract at m/z 981.

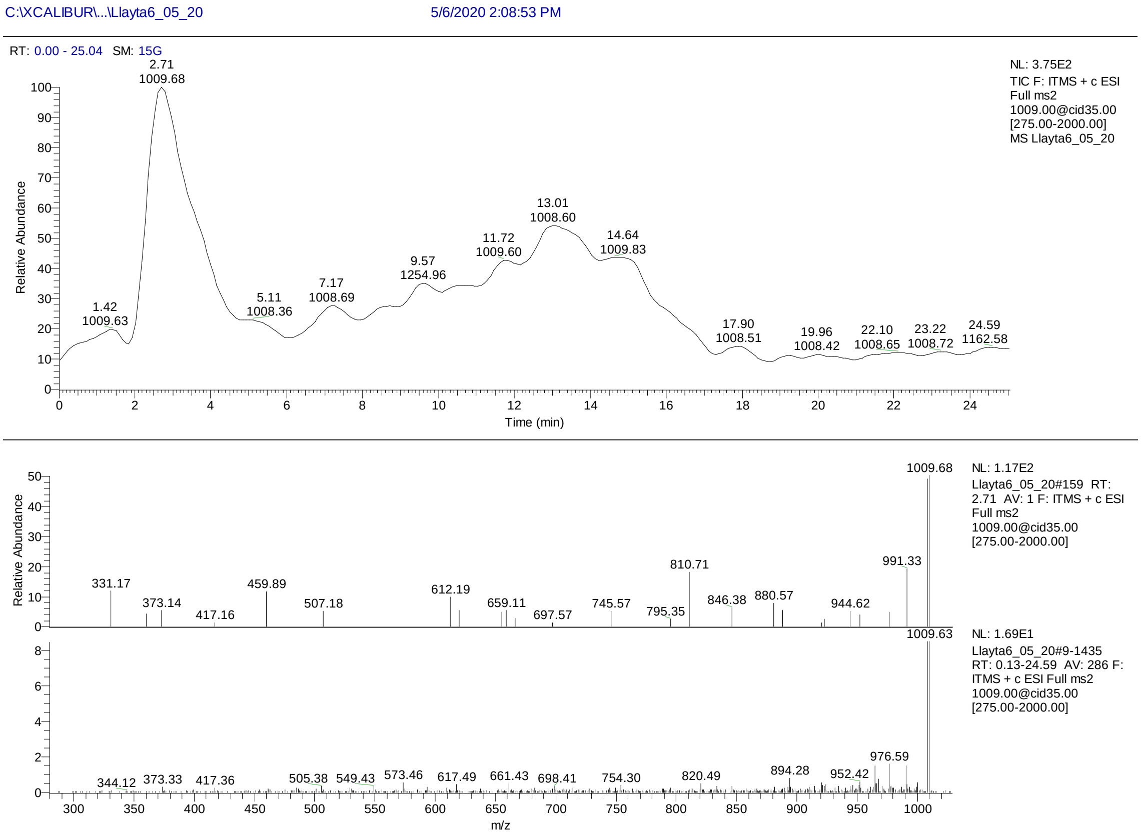

Figure S5. Extracted Ion Chromatogram and Full MS^2 spectra of Llayta extract at m/z 1009.

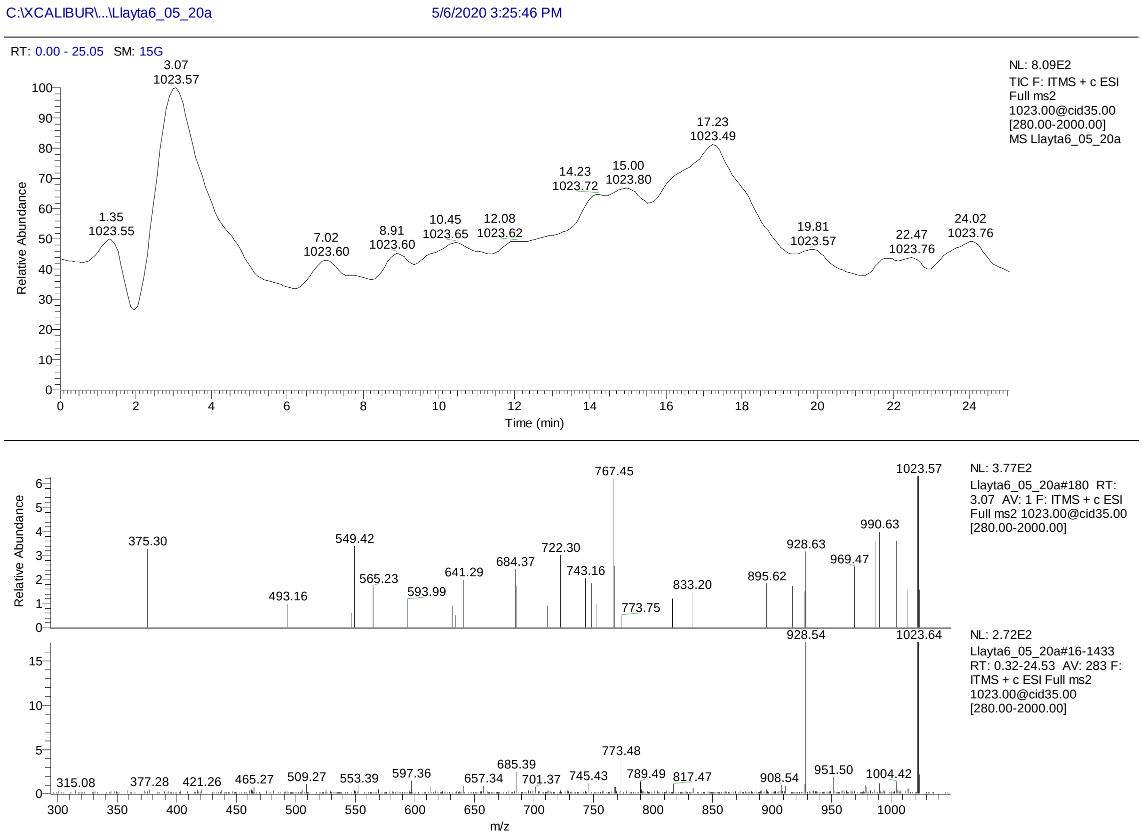

Figure S6. Extracted Ion Chromatrogram and MS² spectra of Llayta extract at m/z 1023.

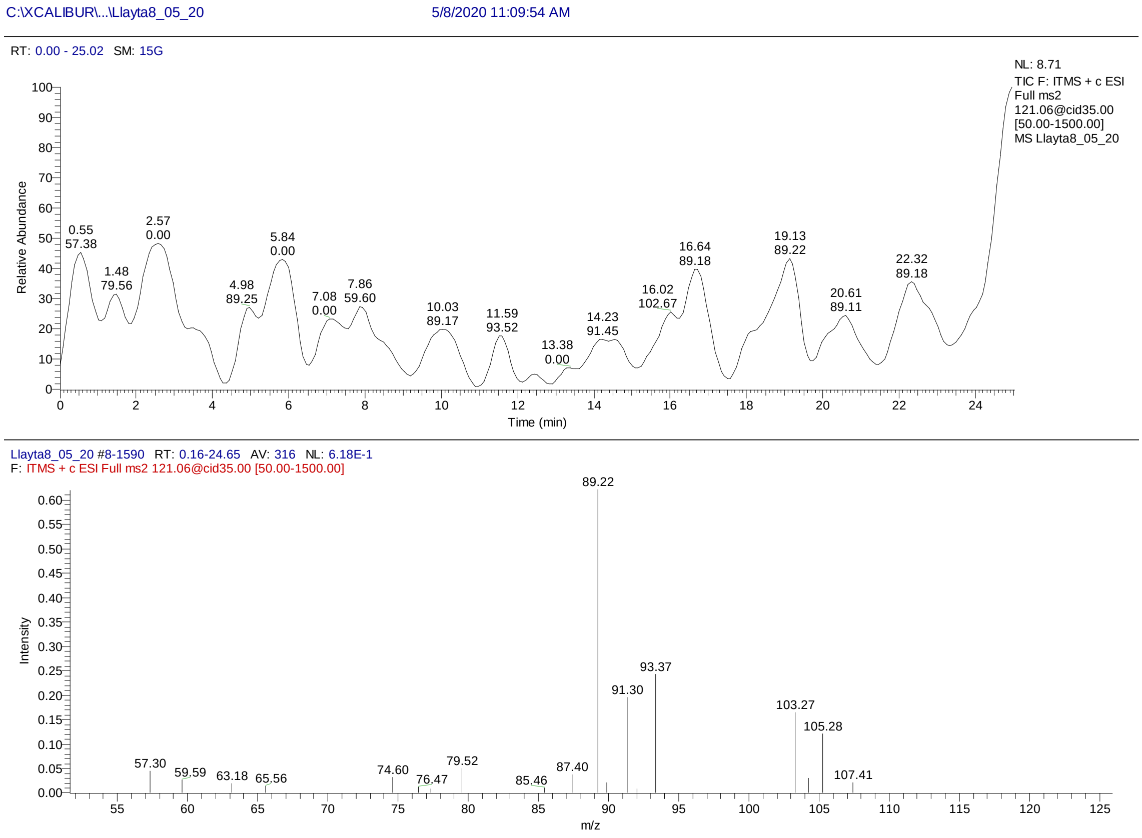

Figure S7. Extracted Ion Chromatrogram and MS² spectrum of Llayta extract at m/z 121.06.

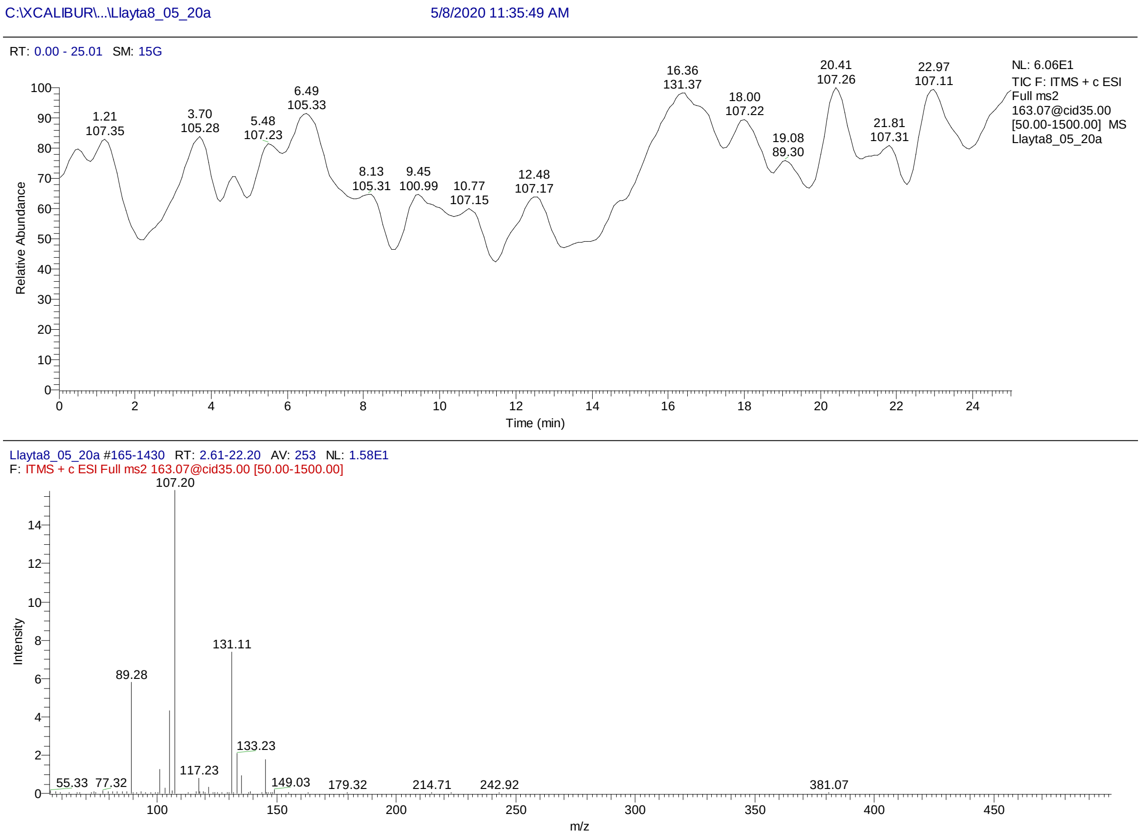

Figure S8. Extracted Ion Chromatrogram and MS^2 spectrum of Llayta extract at m/z 163.07.
